# Transcriptome analysis reveals the impact of arbuscular mycorrhizal symbiosis on *Sesbania cannabina* expose to high salinity

**DOI:** 10.1038/s41598-019-39463-0

**Published:** 2019-02-26

**Authors:** Cheng-Gang Ren, Cun-Cui Kong, Kun Yan, Zhi-Hong Xie

**Affiliations:** Key Laboratory of Biology and Utilization of Biological Resources of Coastal Zone, Yantai Institute of Coastal Zone Research, Chinese Academy of Sciences, 264003 Yantai, China

## Abstract

Arbuscular mycorrhiza can improve the salt-tolerance of host plant. A systematic study of mycorrhizal plant responses to salt stress may provide insights into the acquired salt tolerance. Here, the transcriptional profiles of mycorrhizal *Sesbania cannabina* shoot and root under saline stress were obtained by RNA-Seq. Using weighted gene coexpression network analysis and pairwise comparisons, we identified coexpressed modules, networks and hub genes in mycorrhizal *S. cannabina* in response to salt stress. In total, 10,371 DEGs were parsed into five coexpression gene modules. One module was positively correlated with both salt treatment and arbuscular mycorrhizal (AM) inoculation, and associated with photosynthesis and ROS scavenging in both enzymatic and nonenzymatic pathways. The hub genes in the module were mostly transcription factors including WRKY, MYB, ETHYLENE RESPONSE FACTOR, and TCP members involved in the circadian clock and might represent central regulatory components of acquired salinity tolerance in AM *S. cannabina*. The expression patterns of 12 genes involved in photosynthesis, oxidation-reduction processes, and several transcription factors revealed by qRT-PCR confirmed the RNA-Seq data. This large-scale assessment of *Sesbania* genomic resources will help in exploring the molecular mechanisms underlying plant–AM fungi interaction in salt stress responses.

## Introduction

Excessive soil salinization has become a serious environmental and agronomic issues^1,2^. Soil salinity severely affects crops establishment and growth, leading to substantial production losses. In addition to having developed intrinsic adaptation mechanisms, terrestrial plants have established mutually beneficial relationship with various rhizosphere microorganisms to cope with the negative effects of salinity^3^. One of the most widespread mutualistic plant–microbe relationships is that established with arbuscular mycorrhizal (AM) fungi. Nearly ninety percent of terrestrial plants, including most leguminous plants, are able to establish this type of symbiosis relationships with fungi of the division *Glomeromycota*^4^.

Many studies have found that AM fungi/plant associations make the host plant more tolerant to abiotic stresses^5^. Additionally, AM fungi have be found in hypersaline conditions, and can adapt to these environment^6^. There are reports in the literature that AM fungi enhanced plant salt tolerance and prevented plant yield losses in saline soils^7–10^. They have suggested comprehensive mechanisms to interpret the enhancement in salt tolerance of host plants by AM fungus, such as greater capacity for mineral and water uptake because a larger area of the soil explored by fungal hyphae, better osmotic status^11^ and root hydraulic conductivity^12^, maintenance of lower Na level and higher K^+^/Na^+^ ratios^13^. Nonetheless, few molecular components that allow AM plants to better tolerate salinity have been identified. Also, challenges in the field are that such protective characteristics are commonly regulated by gene network consist of multiple gene families. Hence, any study of the effect of AM symbiosis on the expression of genes with products involved in salt tolerance is complicated by the complexity and multiplicity of the protective characteristics. Nevertheless, many previous works have focused mainly on the physiological characteristics of plants during this beneficial interaction. Very few studies have been reported on transcriptome involved in the AM fungi induced systemic salt tolerance in host plants.

*Sesbania cannabina*, a soil-improving legume is used as green manure to increase the many crops yield in saline soils. Therefore, exploiting *S. cannabina* – AM fungi symbiosis represents a good strategy for reclamation of saline soils. We recently observed that AM fungi could improve salt-tolerance in *S. cannabina* and increase biomass^14,15^. In order to comprehend the underlying mechanisms of mycorrhizal plant response to saline soils, in this study, we investigated the effects of *Funneliformis mosseae* inoculation on the growth of *S. cannabina* exposed to short and long-term salt stress and identified plant genes with key roles in the response to AM fungi by transcriptome profiling in *S. cannabina* shoot and root using Illumina sequencing. We focused on characterization the transcriptome of mycorrhizal plant under salt stress to provide insights into the molecular mechanism that allows AM fungi to induce salt tolerance in plants.

## Results

### Transcriptome sequencing and assembly

In order to confirm the alleviation effect of AM fungi on *S. cannabina* under salinity, plant biomass was measured in the presence or absence of 200 mM NaCl with *F. mosseae* inoculation. In 10 days, the growth of *S. cannabina* inoculated with *F. mosseae* was faster than un-inoculated plants under saline treatment (Fig. S1). Compared with non-inoculated (NM) plants, AM-inoculated (AM) plants exhibited 61.5% and 64.7% increases in shoot and root dry weights in saline conditions, respectively, but the difference was not significant in water-treated groups (Fig. S1b,d). Similar patterns were observed in fresh biomass accumulation (Fig. S1c,e). These results suggested that *F. mosseae* promoted plant growth and biomass under saline conditions.

To understand the mechanisms underlying AM fungi-induced plant salt tolerance, 12 *S. cannabina* RNA-Seq libraries containing two biology replicates for each treatment were constructed from tissues (shoot and root) of AM and NM plants exposed to 200 mM NaCl for 0, 3 and 27 h. The sequencing data were deposited in the NCBI GEO database under accession number GSE99532. Altogether, 1,254,209,094 Illumina paired-end raw reads were generated (Table 1). After adaptor sequences, ambiguous nucleotides and low-quality sequences were removed, 1,199,463,482 clean reads were retained. As shown in Table S1, the clean reads assembly resulted in 202,621 unigenes in the range from 222 to 15,720 bp, with a N50 (2204 bp). The fragments per kilobase of exon per million fragments mapped (FPKM) data were tested by correlation analysis to evaluate sampling between each pair of bioreplicates. The minimum value of all correlation coefficient between each biological pairs was 0.74. (Fig. S2).

**Table 1:** Summary of sequences analysis.

Sample	Raw Reads	Clean reads	Clean bases (Gb)	Error(%)	Q20(%)	Q30(%)	GC(%)
AL1	71017240	69794736	10.47	0.01	97.53	93.84	45.05
AL2	54528464	53599538	8.04	0.01	97.27	93.34	44.2
AR1	59044588	58109962	8.72	0.01	97.32	93.49	43.74
AR2	60546522	59470218	8.92	0.01	97.2	93.24	43.8
ASSL3H1	59932512	59100412	8.87	0.01	97.56	93.9	44.2
ASSL3H2	57653340	56751508	8.51	0.01	97.52	93.79	44.12
ASSR3H1	46719808	45198466	6.78	0.02	96.75	92.11	44.01
ASSR3H2	47367722	45444750	6.82	0.02	96.4	91.61	44.13
ASSL27H1	46241954	44805784	6.72	0.02	96.75	92.03	44.44
ASSL27H2	70153642	67008172	10.05	0.02	96.83	92.07	44.3
ASSR27H1	47129888	45581658	6.84	0.02	96.52	91.74	44.15
ASSR27H2	49307974	46375310	6.96	0.03	94.76	87.97	44.38
L1	51623710	48636508	7.3	0.03	94.77	87.66	44.36
L2	46500884	43656432	6.55	0.03	94.55	87.32	44.44
R1	46891272	43971630	6.6	0.02	96.1	90.69	43.71
R2	48598584	45774804	6.87	0.02	96.43	91.34	43.98
SSL3H1	48885732	45831444	6.87	0.03	94.64	87.73	45.01
SSL3H2	53389098	49955944	7.49	0.03	94.37	87.01	44.91
SSR3H1	45251108	42354266	6.35	0.03	94.61	87.46	44.16
SSR3H2	49960204	46841530	7.03	0.02	94.71	88.05	44.32
SSL27H1	53165568	49679022	7.45	0.03	94.26	86.87	44.48
SSL27H2	49662402	46455738	6.97	0.03	94.33	86.88	44.51
SSR27H1	45342204	42252524	6.34	0.02	95.46	89.51	44.21
SSR27H2	45294674	42813126	6.42	0.02	96.88	92.29	44
Total	1254209094	1199463482	179.94				

R, root; L, shoot; A, AM-inoculated; SS, salt stressd; H, Hour; Q20, The percentage of bases with a Phred value > 20; Q30, The percentage of bases with a Phred value > 30.

### Sequence annotation

As shown in Table 2, the unigenes were searched against several public databases. Among them, 180,065 unigenes (61.88%) had significant matches in Nr, 179,437 (61.66%) in Nt, and 128,079 (44.01%) in Pfam. Altogether, 215,546 unigenes (74.07%) were successfully annotated in at least one of the seven databases and 1505 unigenes (0.51%) annotated in all seven databases.

**Table 2:** BLAST analysis of non-redundant unigenes against public databases.

	Number of Genes	Percentage (%)
Annotated in NR	180065	61.88
Annotated in NT	179437	61.66
Annotated in KO	75476	25.93
Annotated in SwissProt	6939	2.38
Annotated in PFAM	128079	44.01
Annotated in GO	129894	44.64
Annotated in KOG	70231	24.13
Annotated in all Databases	1505	0.51
Annotated in at least one Database	215546	74.07
Total Unigenes	290972	100

**Figure 1:**
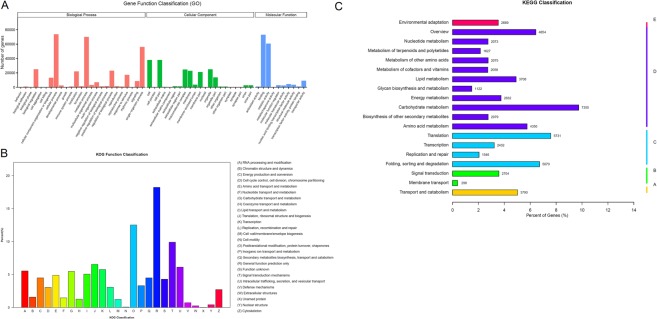
Functional annotation and classfication of *S. cannabina* transcriptome. (**A**), GO categorization of non-redundant unigenes. Each annotated sequence was assigned at least one GO term. (**B**), COG annotation of putative proteins. (**B**), KEGG annotation of putative proteins. The capital letters against the colored bars indicate five main categories, (**A**) cellular processes; (**B**) environmental information processing; (**C**) genetic information processing; (**D**) metabolism; and (**E**) organism systems.

As shown in Fig. 1A, total 129,849 unigenes were subdivided into three domians for GO analysis. In the biological process (BP) group, genes mainly annotated with ‘cellular process’ (73,541), ‘metabolic process’ (69,994), and ‘single-organism process’ (56,048). In the cellular component (CC) group mostly comprised proteins annotated with ‘cell’ (37,972), ‘cell part’ (37,967), and ‘organelle’ (25,008). As in the molecular function (MF) group, the ‘binding’ (72,987), ‘catalytic activity’ (60,633), and ‘transporter activity’ (9045) were predominantly matched terms.

Next, aunigenes were further annotated in the KOG database (Fig. 1B). The results show that 70,231 unigenes were assigned KOG classifications and subdivided into 26 specific groups. The highest number of unigene was belongs to ‘General functional prediction only’ (12798), seconded by ‘post-translational modification, protein turnover, chaperone’ (8788), ‘intracellular trafficking, secretion, and vesicular transport’ (6954), ‘signal transduction mechanisms’ (7613), and ‘translation, ribosomal structure and biogenesis’ (1642). Using the KEGG annotation system, unigene metabolic pathway analysis was also performed. Total of 139 pathways representing 75,476 unigenes (Fig. 1C) were predicted in this process. The pathways involving the largest number of unigene were ‘carbohydrate metabolism’ (7355), seconded by ‘translation’ (5731) and ‘folding, sorting and degradation’ (5070).

### Comparative transcript expression analysis of AM and NM plants in response to salt stress

**Figure 2:**
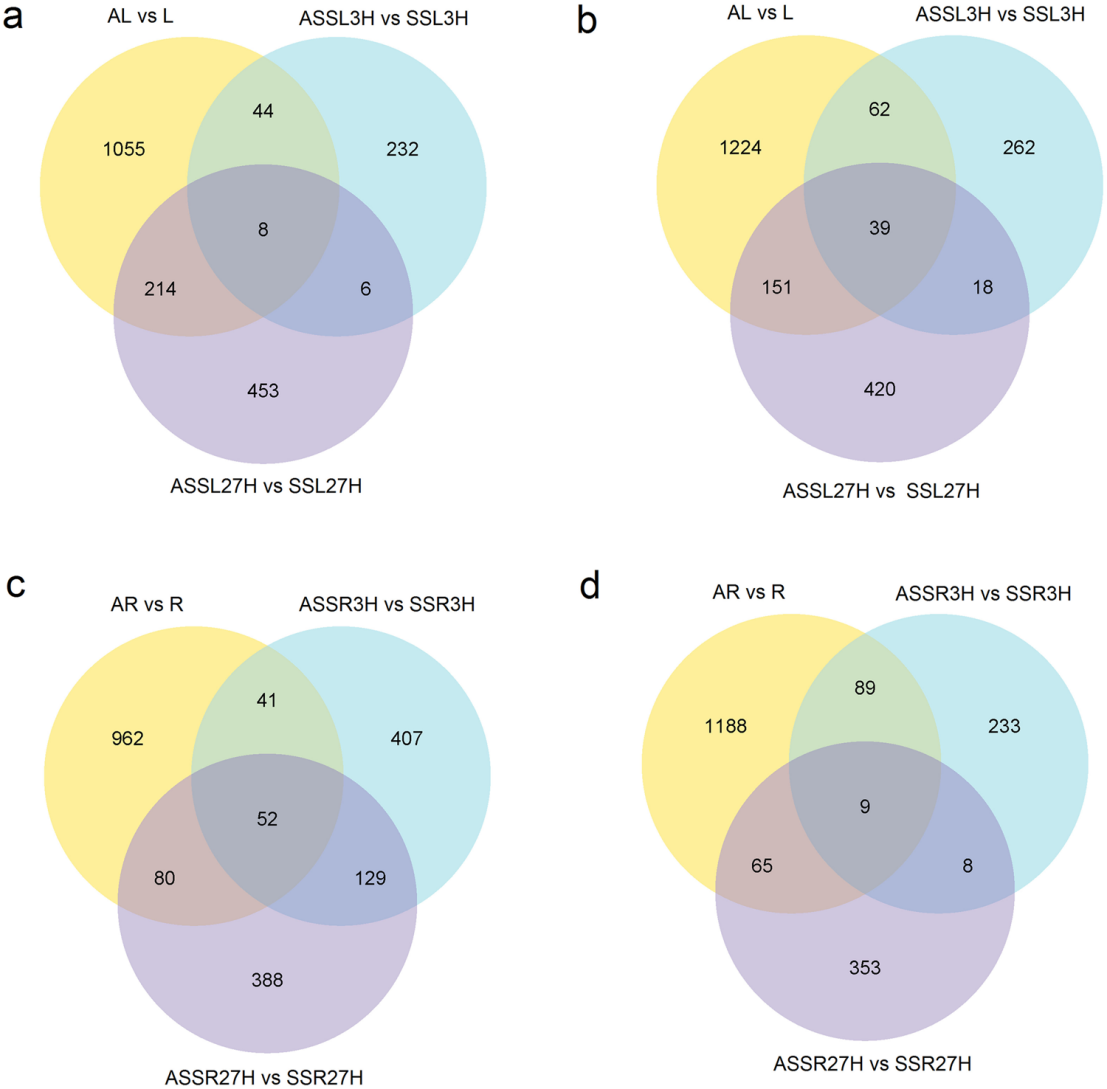
Venn diagrams of differentially expressed transcripts in AM and NM plants under saline treatment in *S. cannabina* plantlets. (**a**), Numbers of DEGs exclusively upregulated in each treatment; (**b**), Numbers of DEGs exclusively downregulated in each treatment. The numbers of DEGs with common or opposite expression change tendencies between treatments are shown in the overlapping regions. The total numbers of up- or downregulated genes in each treatment are the sum of the numbers in each circle.

Differentially expressed genes (DEGs) were defined as unigenes that had significantly enriched or depleted transcripts after the treatments. Based on pairwise comparison, differential expression analysis between AM and NM plants exposed to saline treatment for different times (0, 3 and 27 h) was performed (Table S2). It showed that 2797 and 2486 unigenes, which made up approximately 1.55% and 1.38% of the total unigenes, were differentially expressed in the shoots and roots of AM plants, respectively, compared with NM plants. Moreover, 1188 (0.66%) and 684 (0.38%) transcripts were differentially expressed in the shoots and roots of AM plants, respectively, after 3 h of salt treatment, whilst 1049 (0.58%) and 1200 (0.67%) transcripts were differentially expressed in the shoots and roots of AM plants, respectively, after 27 h of salt treatment. Commonly up- and downregulated unigenes were identified between AM and NM plants in response to short- and longterm salt stress to evaluate the overlap degrees. There were 238 upregulated and 280 downregulated transcripts in AM plant shoots comparing 3 h with CK (Fig. 2a,b), while 459 transcripts were upregulated and 438 transcripts were downregulated in AM plants shoots comparing 27 h with CK (Fig. 2c,d).

In order to reveal co-regulation patterns among the DEGs in mycorrhizal *S. cannabina* in response to short and long periods of saline treatment, the expression profiles of the unigenes were analysis by a hierarchical clustering algorithm. It showed distinct expression patterns for DEGs in AM and NM plants in response to saline treatments (Fig. S3).

### Weighted Gene Coexpression Network Analysis (WGCNA)

**Figure 3:**
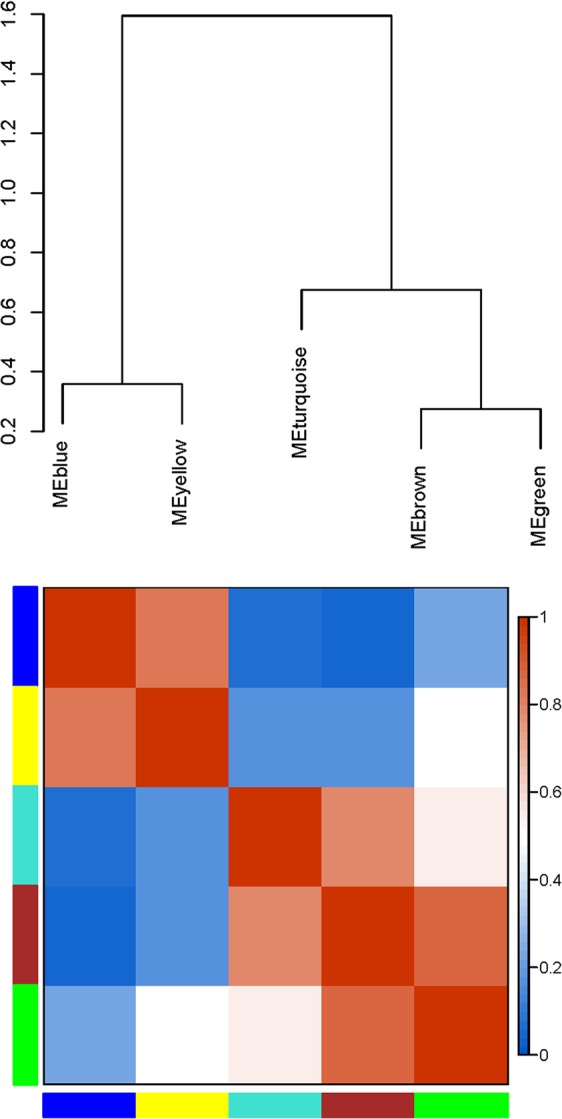
Gene co-expression modules in *S. cannabina* tissues showing the cluster dendrogram constructed based on the eigengenes of the modules (above) and the heatmap for the correlation coefficient between the modules (below).

To explore the acquired salinity tolerance in AM *S. cannabina*, 10,371 genes were selected to construct a scale-free coexpression network using WGCNA. WGCNA is a systemic approach especially for understanding biology networks instead of individual genes. In order to confirm that the network was biologically relevant, the scale-free topology model fit and the mean connectivity of the network was evaluated over a range of soft threshold powers (β), before selecting β = 9 (Fig. S4). A dynamic hierarchical tree algorithm was used to divide the clustering tree constructed from the DEGs, resulting in five coexpression modules, which were labeled turquoise (3,999 genes), blue (2,832 genes), brown (2,328 genes), yellow (676 genes), and green (263 genes) (Figs 3 and S5). The gene cluster modules were enriched in specific GO functional terms, and statistically significantly enriched terms (*p* < 0.05) were selected for further analysis. The genes in the turquoise module were mainly associated with organic substance biosynthetic processes (*p* = 5.1087E-16), organonitrogen compound metabolic processes (*p* = 3.2913E-20), cell parts (*p* = 5.0073E-18), intracellular regions (*p* = 9.10E-13), and structural molecule activity (*p* = 2.7081E-60); genes in the blue module were mainly involved in oxidation-reduction processes (*p* = 8.1242E-28), single-organism metabolic processes (*p* = 8.40E-19), membrane protein complexes (*p* = 0.028578), oxidoreductase activity, acting on other nitrogenous compounds as donors, with NAD or NADP as acceptor (*p* = 0.032091), metal ion binding (*p* = 0.000449) and catalytic activity (*p* = 4.03E-11); genes in the brown module were involved in single-organism metabolic processes (*p* = 0.000863) and oxidoreductase activity (*p* = 0.012702); the genes of the yellow module were involved in organic substance catabolic processes (*p* = 9.97E-05), lipid metabolic processes (*p* = 0.001364) and hydrolase activity, acting on glycosyl bonds (*p* = 0.000244); and genes of the green module were mainly involved in trehalose biosynthetic processes (*p* = 0.036706), disaccharide biosynthetic processes (*p* = 0.036706) and oligosaccharide biosynthetic processes (*p* = 0.64995).

At last, the results of eigengene–trait correlation analysis indicated that the blue module was positively correlated with both salt stress and AM inoculation after multiple testing correction (*p* < 0.05) (Fig. S6). Identifying the genes in this module and their biological roles in response to salinity and AM inoculation was of particular interest, so functional annotation was performed. Detailed information on the GO enrichment in the modules is provided in Table S3 with *P*-value < 0.05. According to it, the blue module was significantly enriched with unigenes functioning in oxidation-reduction processes, oxidoreductase activity, photosynthetic membranes, and iron ion binding. These biological activities may play central roles in mycorrhizal plants under saline stress. KEGG pathways (http://www.genome.jp/kegg/) enrichment analysis (Table S4) showed that photosynthesis, carotenoid biosynthesis and glycine, serine, alanine, aspartate and glutamate metabolism were the most enriched physiological processes.

**Figure 4:**
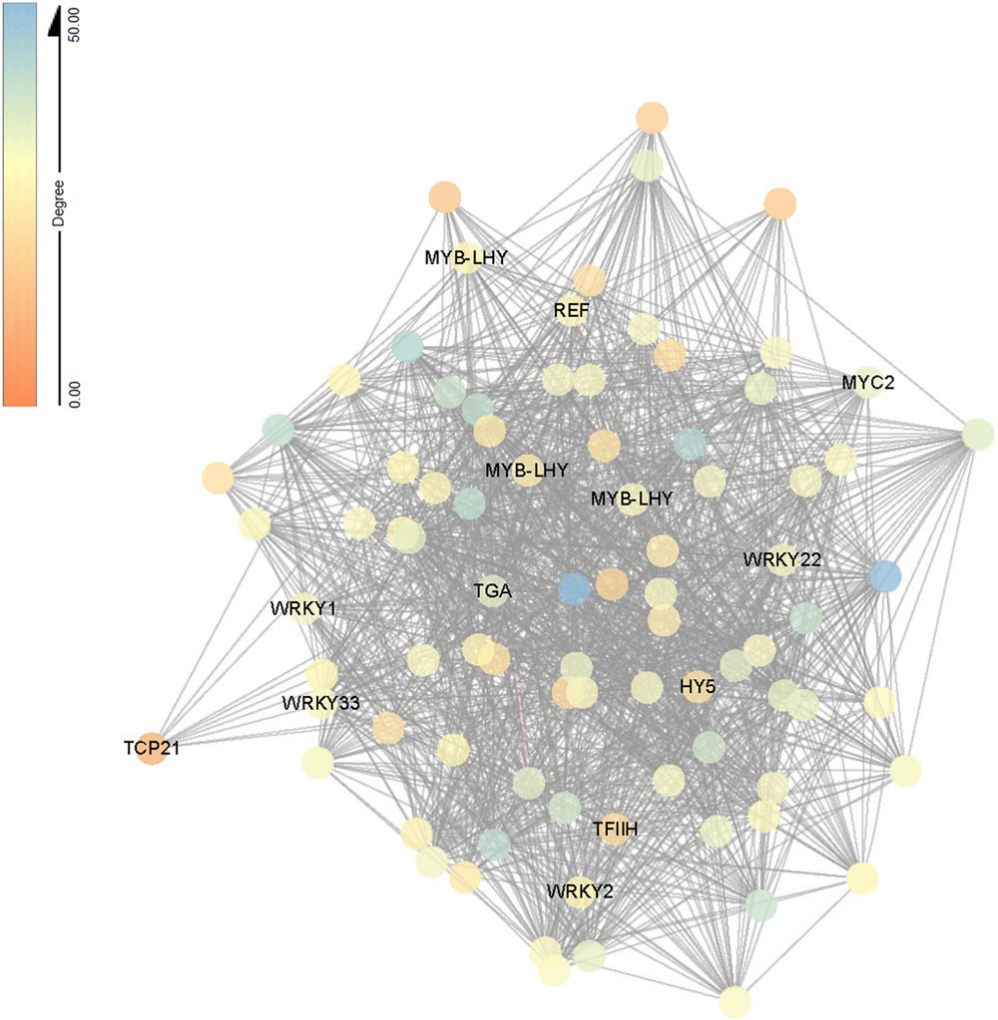
Network visualization of interactions between hub genes in bule module. Transcription factors are labeled with name. Eighty-five genes (top 3% of hub) are visualized by Cytoscape.

Hub genes are genes which have the most connections in a network. As shown in the blue module, 34 of the 85 hub genes (the top 3%) encoded transcription factors (Tables S5 and S6). The majority of transcription factors belonged to the MYB family, followed by the WRKY, ethylene-responsive transcription factor and circadian regulator TCP families. Besides genes encoding transcription factors, photosystem I subunit (Cluster-45083.137562, Cluster-45083.138788, Cluster-45083.140135), ribose 5-phosphate isomerase A (Cluster-45083.140006), light-harvesting complex II chlorophyll a/b binding protein (Cluster-45083.139493, Cluster-45083.138788) and peroxidase (Cluster-45083.145260) genes had the highest connectivity in the network (Fig. 4; Table S5).

### Validation of RNA-Seq data by qRT-PCR

To verify our bioinformatics analyses, the expression levels of 12 selected genes which encode WRKY transcription factor, MYB-related transcription factor LHY, ethylene-responsive transcription factor, transcription factor TCP, photosystem I subunit VI, ribose 5-phosphate isomerase A, light-harvesting complex II chlorophyll a/b binding protein, copper/zinc superoxide dismutase (SODC), nickel-containing superoxide dismutase, catalase, glutathione peroxidase, peroxidase were quantified by qRT-PCR. The results showed comparable expression patterns of all the selected genes in real-time PCR analysis and in the RNA-Seq data (Fig. S7a,b). The correlation between RNA-Seq and qRT-PCR platforms was also calculated using SPSS V17.0 (SPSS, Inc., Chicago, IL, USA), the pearson correlation coefficients were 0.903 and 0.939 (Fig. S7c), confirming that our experimental results were valid.

## Discussion

It has been proposed that mycorrhizal colonization enhances plant salt tolerance by improving photosynthetic ability, water and nutrient uptake, ion balance and osmolyte concentrations, among other characteristics^16–18^. However, information about the molecular bases of such effects is limited. The present study demonstrated that *F. mosseae* alleviated salinity stress in *S. cannabina* as shown in previous studies. Illumina sequencing and WGCNA analysis were used to reveal the gene network stimulated by AM fungi in *S. cannabina* plantlets under salt treatment.

Improvements in photosynthetic activity have been reported in mycorrhizal plants growing under saline conditions. It has been found that AM inoculation improved the photosynthetic efficiency of maize, primarily though increasing gas exchange capacity and the PSII efficiency and regulating the energy distribution between photochemical and non-photochemical processes^19^. Similar conclusions were drawn by Lin *et al*.^20^ in experiments with *Leymus chinensis* seedlings. Another study showed that AM inoculation enhanced photosynthetic efficiency by improving stomatal conductance and protecting photochemical events of PSII against salt stress^10^. In this study, photosynthesis was one of the most enriched pathways and included genes related to photosystem II, photosystem I and the light-harvesting chlorophyll–protein complex (Fig. S8a,b; Table S4). Enzymes involved in carbon assimilation such as 5-bisphosphate carboxylase/oxygenase (Rubisco) also play a key role in photosynthesis process^21^. Mo *et al*.^22^ discovered that AM inoculated watermelon seedlings had higher water-use efficiency and Rubisco activity than NM seedlings under drought treatment. In contrast, data about Rubisco enzyme in AM plants under salt treatment is limited. The present study showed genes related to carbon fixation in photosynthetic organisms were also significantly enriched (Fig. S8c; Table S4). All of the above, plus we found that AM inoculation increasing photosystem II efficiency (ΦPSII) values, carbohydrate content, and the activities of ADP-glucose pyrophosphorylase (AGPase) and starch synthase, and a decrease in non-photochemical quenching of chlorophyll fluorescence (NPQ) (Fig. S9), led to the conclusion that AM fungi enhance salinity tolerance of *S. cannabina* by maintaining photosynthetic ability.

Salinity stress can induce the formation of ROS, its excessive accumulation cause oxidative stress^23^. Several reports have proved that AM inoculation enable host plants to tolerate saline stress by elevating the activities of antioxidant enzymes including superoxide dismutase (SOD), catalase (CAT), glutathione reductase (GR), and peroxidase (POX)^16,24–26^. In the current study, the most enriched GO functional term (*p* < 0.05) in the module that was positively correlated with both saline treatment and AM inoculation was ‘oxidation-reduction process’. Some of the unigenes were verified by qRT-PCR, and the increased expression levels of genes related to SOD, CAT, GR and POX in AM plants compared with NM plants indicated that AM fungi enhanced the ROS-scavenging capacity in *S. cannabina* plants. To date, the studies about effects of AM inoculations on nonenzymatic antioxidants accumulation in the host plant are limited. Ascorbate is the most important antioxidant in plants and, in association with other components of the antioxidant system, protects plants against oxidative damage resulting from aerobic metabolism and photosynthesis. Carotenoids function as photoprotectants by quenching ROS before oxidative damage can occur, or by active non-photochemical quenching (NPQ), or heat dissipation, of excess light energy^27,28^. Our data showed that pathways associated with ascorbate and aldarate metabolism and carotenoid biosynthesis were significantly enriched (Fig. S10; Table S4), which could reflect key mechanisms of acquired systemic salt tolerance in AM *S. cannabina*.

To sum up, this study showed that tolerance of *S. cannabina* to salinity stress was improved by AM inoculation. In order to identify genes essential in the acquired salinity tolerance of AM plants, we investigated transcriptome data from *S. cannabina* tissues under salt stress using Illumina sequencing and WGCNA analysis. This analysis revealed one coexpression module that responded to salt stress and AM inoculation in shoots. The results showed that genes related to photosynthesis, ROS scavenging and specific transcription factors played central roles in the plant salt tolerance acquired through AM symbiosis. Our work may facilitate plant functional genomic studies, such as by suggesting candidate genes as potential markers of tolerance to salt stress.

## Materials and Methods

### Plant material and treatments

*S. cannabina* (Retz.) Pers. seeds from a single population (Shandong Academy of Agricultural Sciences, China) were surface sterilized (10% hydrogen peroxide, 10 min) before use. The seeds were pregerminated and sown in autoclaved zonolite. *Glomus mosseae* (BGC NM03D) was propagated with *Trifolium repens* plants for 60 days, infected roots, hyphae, spores and substrate was used as inoculum. After 2 weeks, each seedlings were transferred to a PVC pots (1 L) with a partition in the middle, and the roots of each seedling were divided and buried on both sides of the partition with autoclaved zonolite; on one side the roots were inoculated with 10 g inoculums containing approximately 121 spores. *S. cannabina* plants were cultured in 12 h photoperiod, 25/17 °C (day/night), and 600 μmol m^−2^ s^−1^ light intensity. Three-week-old plantlets were used for all treatments with 200 mM NaCl solution. The 27-h group was treated first and then the 3-h group was treated 24 h later. After the treatments were performed, all samples from the three treatments (0, 3, and 27 h) were harvested at the same time point, frozen immediately in liquid N_2_ and stored at −80 °C before use.

### Quantification of Arbuscular mycorrhizal fungal colonization

Percentage of mycorrhizal roots colonization was measured according the gridline intersection method after samples were stained with trypan blue as previously described^14^.

### RNA isolation and sequencing

Total RNA extraction and purification, and cDNA library construction were performed as previously described^29^. Samples from three seedlings were pooled for a single biological replicate; total RNA from two biological replicates was isolated. RNA-Seq library was constructed using the NEBNext Ultra Directional RNA Library Prep Kit for Illumina (NEB, Ispawich, USA). Then, Illumina HiSeq. 4000 platform were used for RNA-Seq libraries sequencing, 150-bp paired ended reads were generated. It was carried out by Novogene Bioinformatics Technology Co. Ltd (Tianjing, China).

### Assembly and annotation

The clean reads were obtained by removing low-quality regions and adapter by NGS QC Toolkit (v2.3)^30^, for all following analyses. Trinity was used for transcriptome assembly with min_kmer_cov set to 2 by default^31^. Functional annotation of unigene were based on seven public databases, Nr (NCBI non-redundant protein sequences), Nt (NCBI non-redundant nucleotide sequences), Swiss-Prot (a manually annotated and reviewed protein sequence database), KOG/COG (Clusters of Orthologous Groups of proteins), KO (KEGG Ortholog database), Pfam (Protein family) and GO (Gene Ontology), using the Blast2GO software with a cutoff E-value of 10^−6^.

### Quantification and differential analysis of gene expression levels

First, gene expression levels were estimated by RSEM^32^, the clean data were mapped back onto the transcriptome assembly using bowtie2 by default (mismatch 0). The read count for unigenes was obtained and normalized to fragments per kilo base of transcript sequence per millions base pairs sequenced (FPKM). Second, differential expression between two treatments was analyzed using the DESeqR package (1.10.1)^33^. The *P*-values were adjusted by Benjamini’s approach^34^ for controlling the false discovery rate (FDR); FDR adjusted *P* < 0.05 and absolute value of log2 ratio > 1 were set as the threshold for significant differential expression. GO enrichment analysis of DEGs was carried out using the GOseq R package based on Wallenius’ non-central hyper-geometric distribution^35^, which can adjust for gene length bias in DEGs. The statistical enrichment of DEGs in KEGG pathways^36,37^ was performed by the KOBAS software.

### Construction and Visualization of Co-expression network

Gene co-expression networks were constructed by WGCNA package (v1.29) in R^38^. Genes with averaged NRPKM values from two replicates > 2 were used for the WGCNA unsigned network construction, and the average NRPKM values were imported into WGCNA. Modules were obtained using the automatic network construction function blockwiseModules with default settings, except that the power of nine was chosen to make the networks showed an approximate scale-free topology (model fitting index *R*^2^ = 0.9), TOM-Type was signed, minModuleSize was 100, and merge Cut Height was 0.25. The edge weight (from 0 to 1) of any two genes was determined by the topology overlap measure provided in WGCNA. Connectivity was defined as the sum of the weights across all the edges of a node, and the top 3% of genes with the highest connectivity in the network were defined as hub genes^39^.

To identify modules that were significantly associated with the acquired salt tolerance in AM plants, the module eigengene was calculated in each module, and then correlated with each sample (saline treatments, AM fungi inoculation and tissues). Only modules with *P* < 0.05 were considered related modules. Coexpression patterns and interactions of hub genes were visualized using Cytoscape^40^.

### qRT-PCR analysis

Total RNA was extracted from shoots using TRIzol (Invitrogen, Carlsbad, CA, USA) following the manufacturer’s instructions, with three biological replicates each sample. The reverse transcription reactions were performed using the GoScript™ Reverse Transcription System (Promega, Madison, WI, USA). The quantitative real-time (qRT)-PCR analysis was conducted with an iCycler iQ real-time PCR detection system (BIO-RAD). Each reaction was completed in a total volume of 20 μl including 0.2 μM primers, 2 μl diluted cDNA, and 10 μl 2 × SYBR Green PCR Master Mix (TaKaRa Bio Inc., Dalian, China). Gene-specific primers were showed in Table S5. Actin and tubulin genes were used for normalization (Table S7). Relative gene expression levels of each gene were calculated using the 2^−△△^*C*t method.

### Photosynthetic parameters

Gas exchange and modulated chlorophyll fluorescence parameters were simultaneously detected using a LI-6400XTR Portable Photosynthesis System equipped with a Fluorometer (Li-Cor, Lincoln, NE, USA). Efficiency of Photosystem II (ΦPSII) and non-photochemical quenching of chlorophyll fluorescence (NPQ) were calculated according to a previously described method^15^.

### Measurement of total carbohydrate content

Root tissue was used to measure the total carbohydrate content according to previously described method^29^. In brief, non-structural carbohydrates were extracted from 100 mg non-AM root tissues. The extract was hydrolyzed by adding HCl (3 M) boiling for three hours. Then it was neutralized by adding Na_2_CO_3_. Total carbohydrate content was measured using anthrone-sulfuric acid colorimetric method, the absorbance was monitored at 630 nm.

### Measurement of enzyme activities

The extraction and investigation of the activities of the AGPase and starch synthase were performed as previously described^29^, with three replicates for each treatment.

## Supplementary information

Transcriptome analysis reveals the impact of arbuscular mycorrhizal symbiosis on Sesbania cannabina expose to high salinity

Supplementary Dataset 1

Supplementary Dataset 2

Supplementary Dataset 3

Supplementary Dataset 4

Supplementary Dataset 5

Supplementary Dataset 6

Supplementary Dataset 7

## Data Availability

Raw sequencing data, as well as the assembly was deposited in NCBI GEO database under accession number GSE99532. All data generated or analysed during this study are included in this published article [and its supplementary information files].
