# Supplementary material for: Transcriptome analysis reveals the impact of arbuscular mycorrhizal symbiosis on Sesbania cannabina expose to high salinity
Source: Sci Rep. 2019 Feb 26;9:2780. doi: 10.1038/s41598-019-39463-0 (PMC6391373; doi:10.1038/s41598-019-39463-0)
Supplement: Supplementary file 1 — Transcriptome analysis reveals the impact of arbuscular mycorrhizal symbiosis on Sesbania cannabina expose to high salinity [file 41598_2019_39463_MOESM1_ESM.pdf]

Supplementary files:

Transcriptome analysis reveals the impact of arbuscular mycorrhizal symbiosis on *Sesbania cannabina* expose to high salinity

Authors:

Cheng-Gang Ren, Cun-Cui Kong, Kun Yan, Zhi-Hong Xie

Corresponding author: Zhi-Hong Xie

Email: zhxie@yic.ac.cn

Telephone: +86-0535-2109183

Supplementary figures:

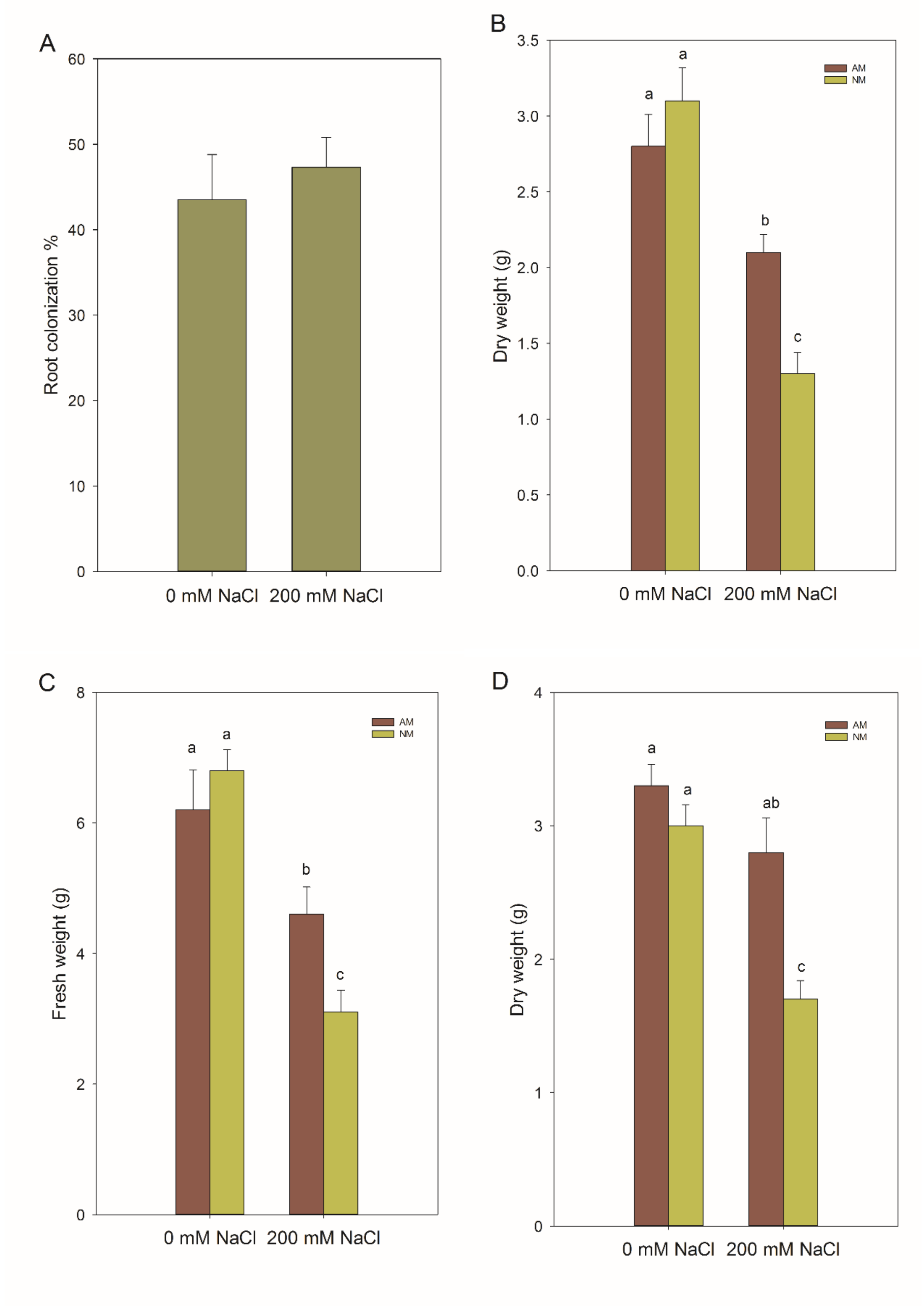

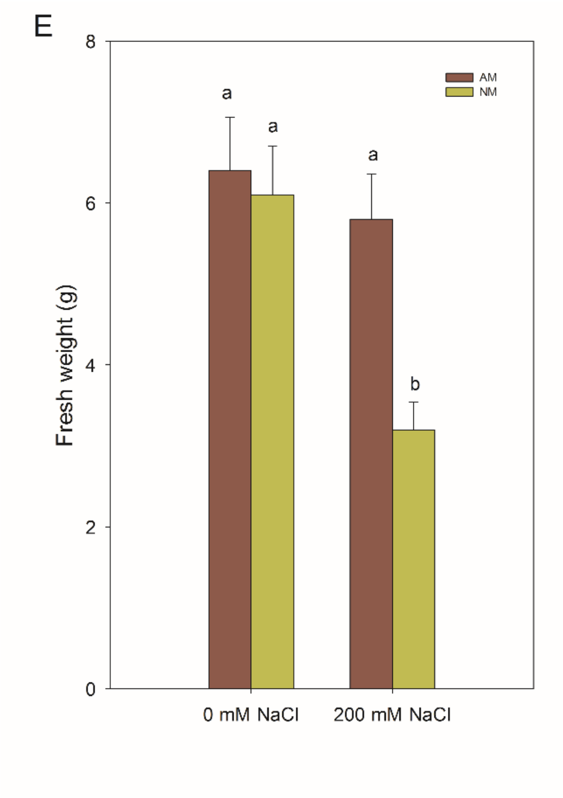

Supplementary figure S1: Impact of AM symbiosis on plant fitness under saline stress. A, The rate of fungal colonization on *S. cannabina* roots with or without NaCl treatment after 10 days. B, Dry weight of shoot were measured for AM and NM plants. C, Fresh weight of shoot were measured for AM and NM plants. D, Dry weight of root were measured for AM and NM plants. E, Fresh weight of root were measured for AM and NM plants.

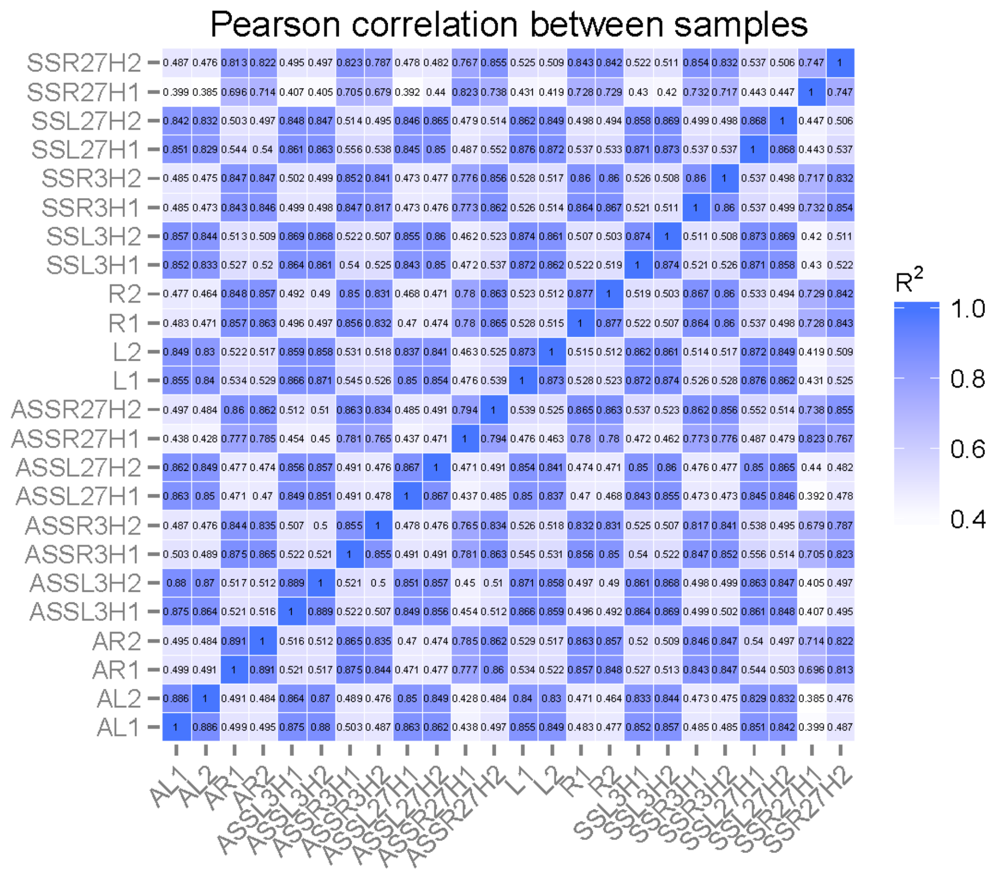

Supplementary figure S2: Distribution of correlation co-efficiencies between each pair of samples.

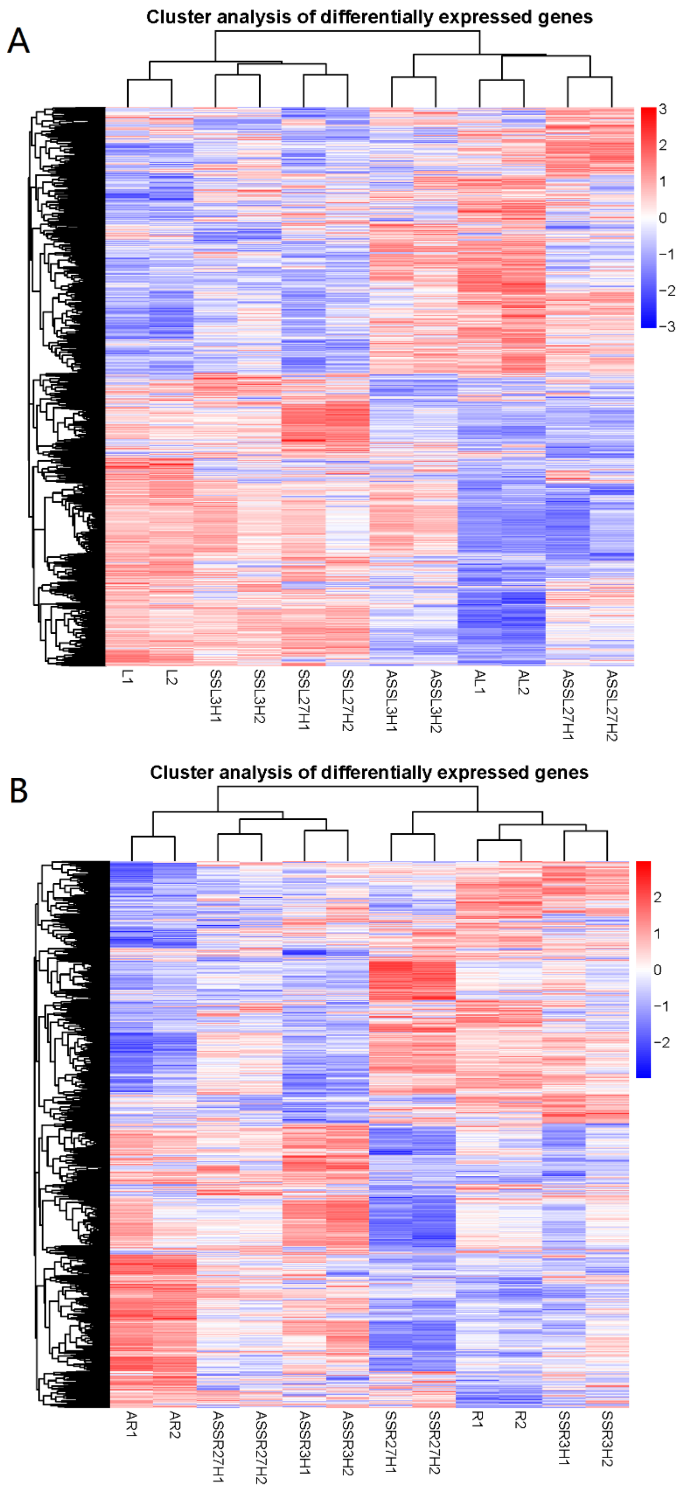

Supplementary figure S3: Cluster analysis of the transcriptome of AM and NM *S. cannabina* plantlets subjected to salt stress. (A) Cluster analysis of the transcriptome of shoots. (B) Cluster analysis of the transcriptome of roots. Each column represents a sample and each line represents a single gene. Different colors indicate different expression levels. Red indicates upregulation and green indicates downregulation, whereas black indicates unchanged expression.

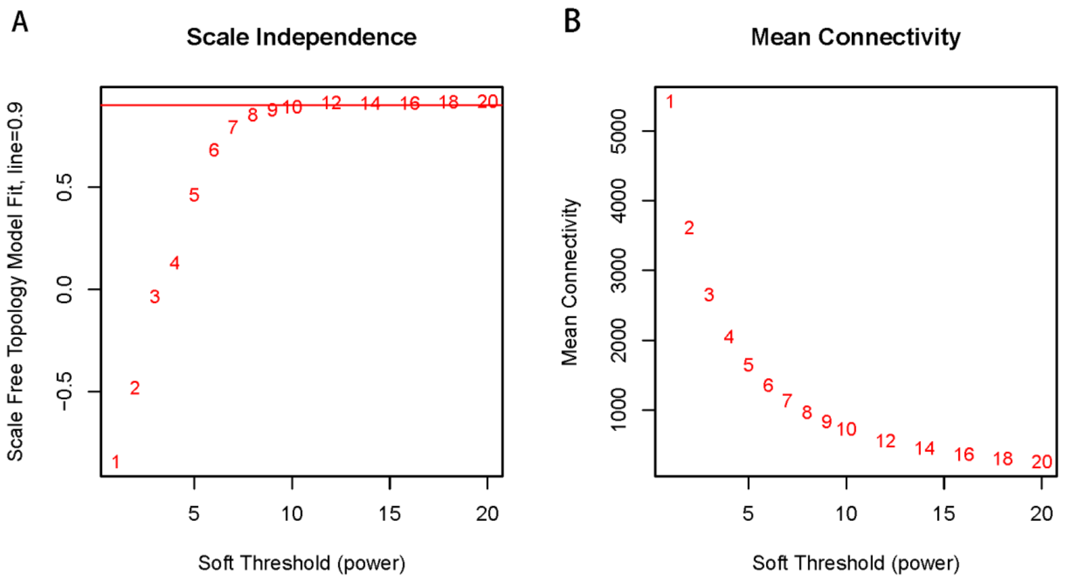

Supplementary figure S4: Scale free topology model fitting by trimmed data. (A) Plot showing the scale free topology R^2 values (y-axis) in function of increasing soft thresholding power (x-axis). (B) Plot showing the relation between mean connectivity (degree, y-axis) and soft threshold (x-axis).

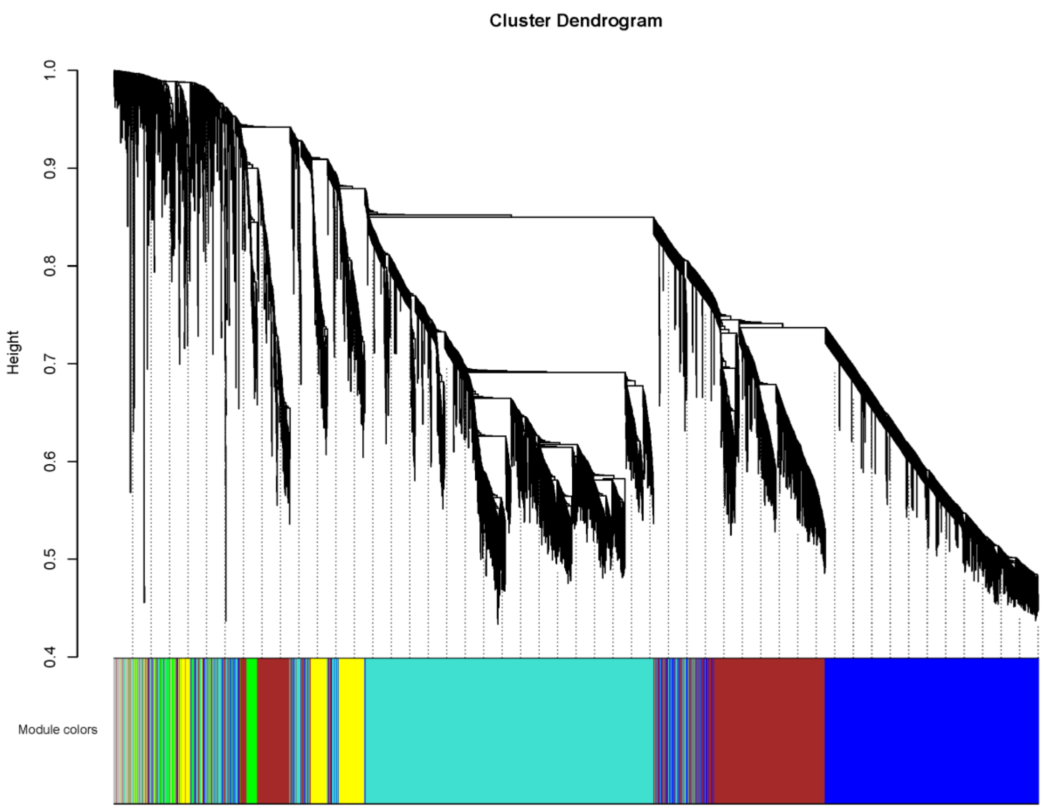

Supplementary figure S5: WGCNA of genes in *S. cannabina* tissues. Hierarchical cluster tree showing coexpression modules identified by WGCNA. Each leaf in the tree is one gene. The major tree branches constitute 5 modules labeled by different colors.

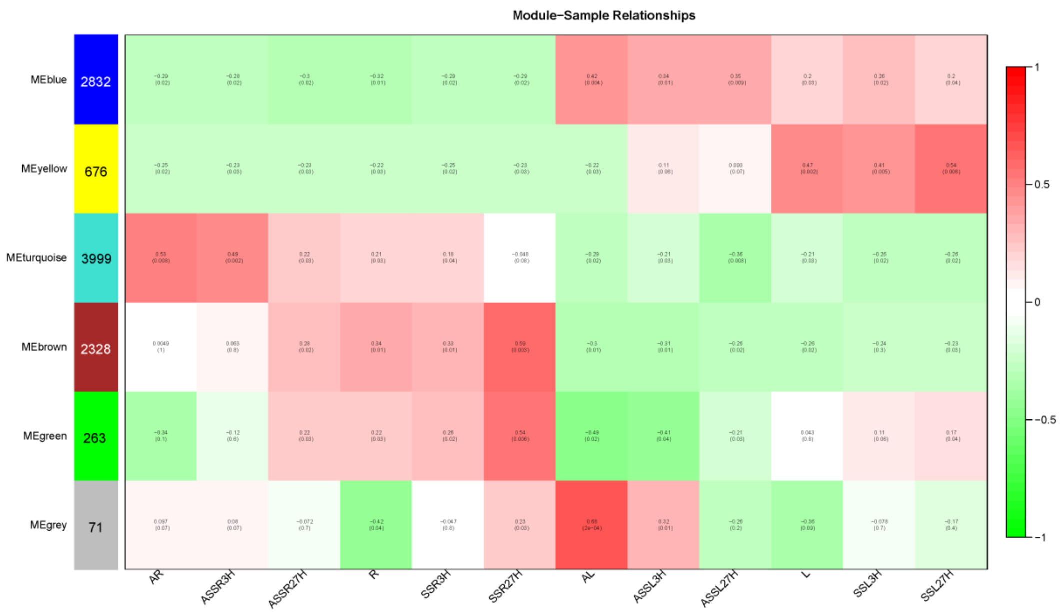

Supplementary figure S6: Module-sample association. Each row corresponds to a module. The number of genes in each module is indicated on the left. Each column corresponds to a specific tissue and treatment. Each cell contains the corresponding correlation and p value. The table is color-coded by correlation according to the color legend.

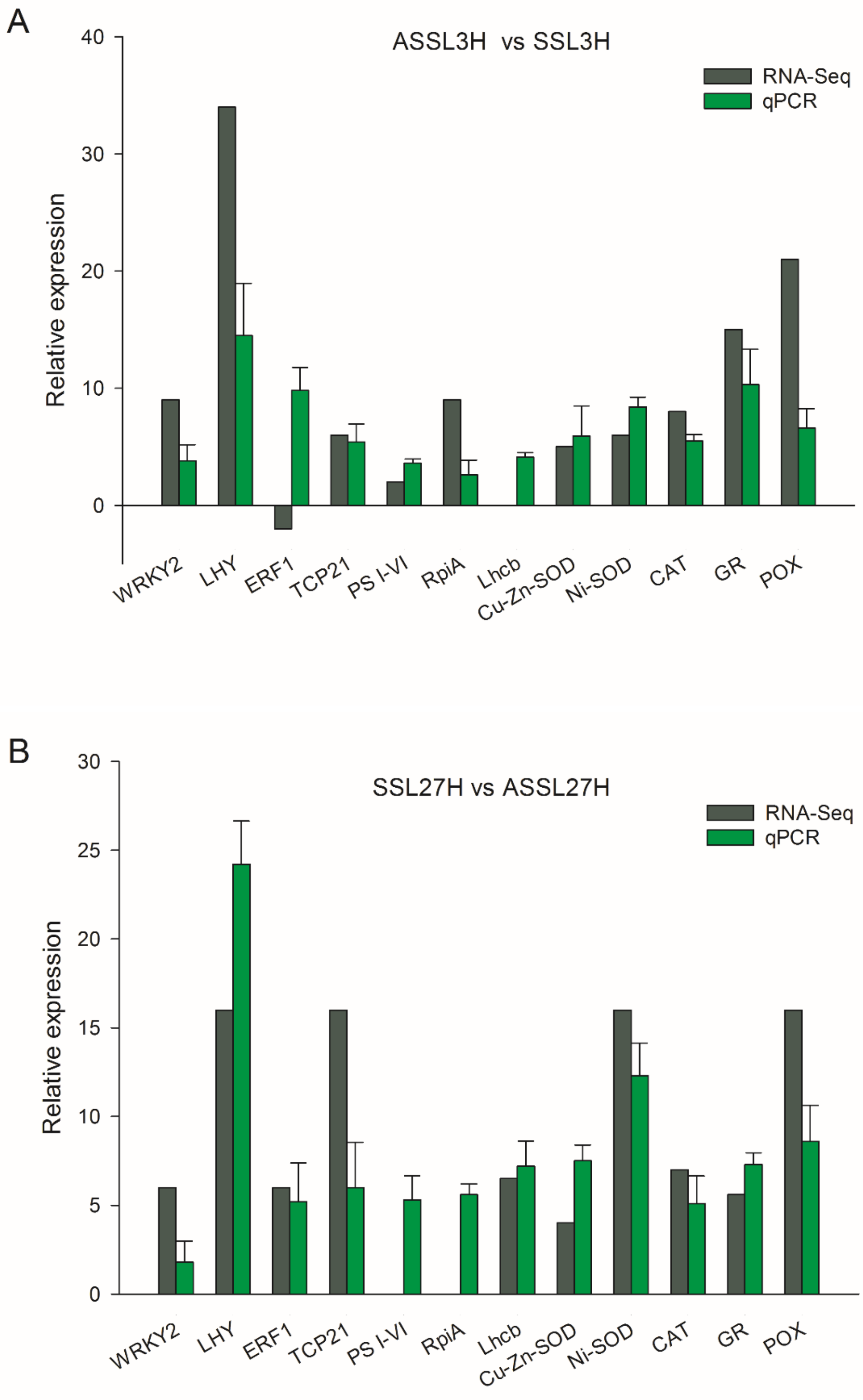
A
ASSL3H vs SSL3H
RNA-Seq
qPCR
Relative expression
40
30
20
10
0
WRKY2
LHY
ERF1
TCP21
PS I-VI
RpiA
Lhcb
Cu-Zn-SOD
Ni-SOD
CAT
GR
POX
B
SSL27H vs ASSL27H
RNA-Seq
qPCR
Relative expression
30
25
20
15
10
5
0
WRKY2
LHY
ERF1
TCP21
PS I-VI
RpiA
Lhcb
Cu-Zn-SOD
Ni-SOD
CAT
GR
POX

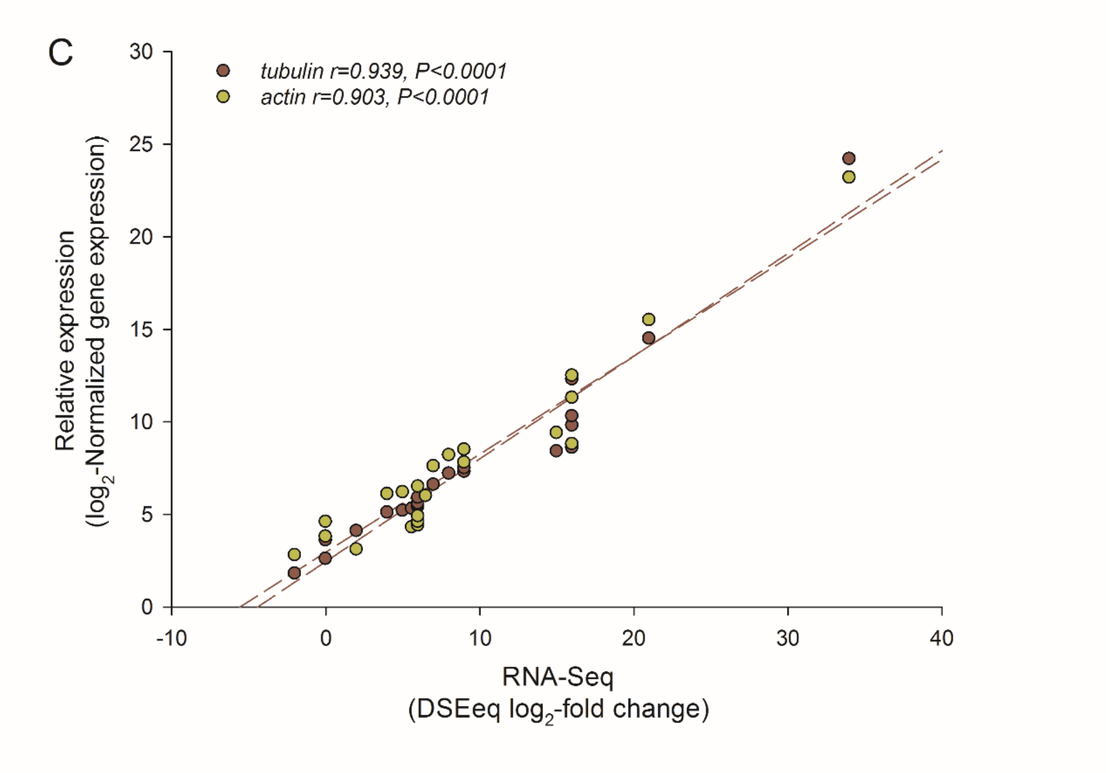

Supplementary figure S7. Validations of selected DEGs during waterlogging and comparison of expression profile by RNA-Seq and qRT-PCR

(A) The relative expression of DEGs between AM and NM plant under 3 h saline treatment. (B) The relative expression of DEGs between AM and NM plant under 27 h saline treatment. Relative expression was analyzed by the $2^{-\Delta\Delta CT}$ method. The results of qRT-PCR are expressed as the mean ±standard deviation (±SD) of at least three replicates. Cluster-45083.140116 was annotated as WRKY transcription factor 2, WRKY2, Cluster-45083.138587 as MYB-related transcription factor LHY, LHY, Cluster-39554.0 as ethylene-responsive transcription factor 1, ERF1, Cluster-45083.236039 as transcription factor TCP21, TCP21, Cluster-45083.137562 as photosystem I subunit VI, PS I-VI, Cluster-45083.140006 as ribose 5-phosphate isomerase A, RpiA, Cluster-45083.139493 as light-harvesting complex II chlorophyll a/b binding protein, Lhcb, Cluster-45083.142570 as Copper/zinc superoxide dismutase, Cu-Zn-SOD, Cluster-45083.94812 as Nickel-containing superoxide dismutase, Ni-SOD, Cluster-45083.134446 as Catalase, CAT, Cluster-45083.126381 as glutathione peroxidase, GR, and Cluster-45083.145260 as peroxidase, POX. (C) Comparison of expression profiles of 12 selected genes by RNA-Seq and RT-qPCR showing different expression profiles. Each data point represent the log2 normalized expression level obtained from RNA-Seq (y axis) and qRT-PCR (x axis) analyses. S. cannabina actin and tubulin gene were used as normalizer.

A

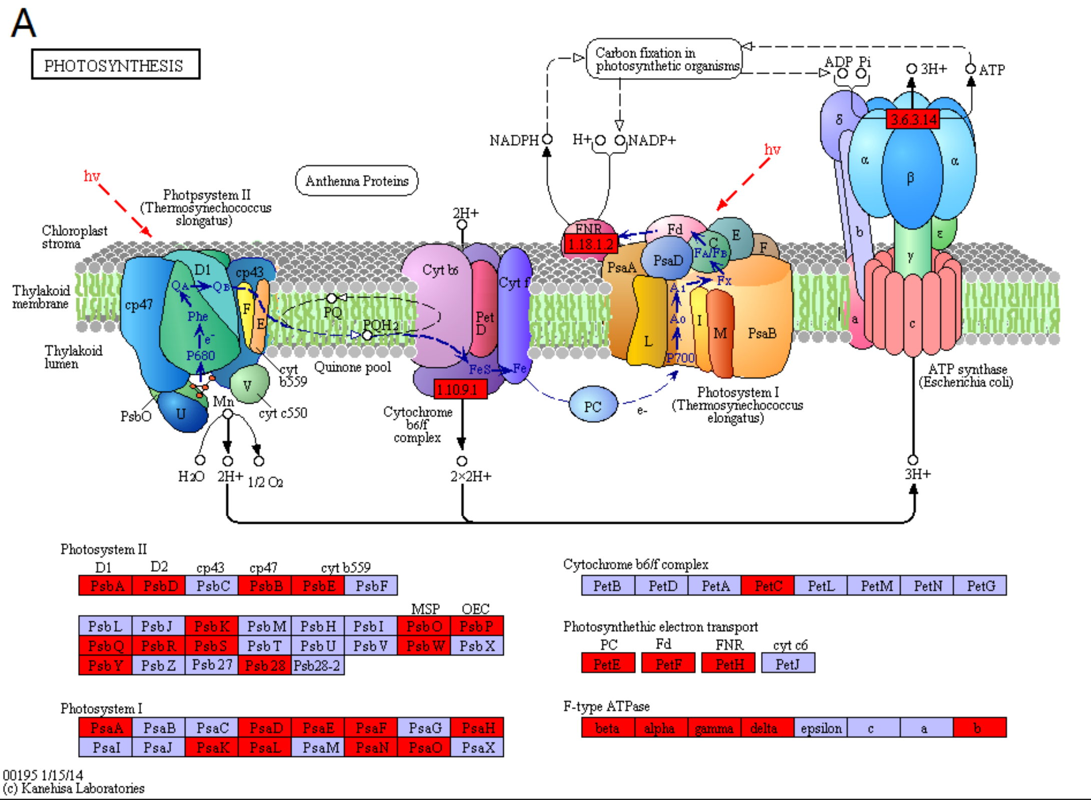
PHOTOSYNTHESIS
Carbon fixation in photosynthetic organisms
ADP Pi
3H+
ATP
NADPH
H+
NADP+
hv
Photpsystem II (Thermosynechococcus slongatus)
Anthenna Proteins
Chloroplast stroma
Thylakoid membrane
Thylakoid lumen
2H+
FNR
1.18.1.2
Fd
3.6.3.14
D1
cp43
cp47
PQ
PQH2
Quinone pool
cyt b559
cyt c550
PsbO
U
V
Mn
H2O
2H+
1/2 O2
Cyt b6
Cyt f
Pet D
1.10.9.1
Cytochrome b6/f complex
2×2H+
PC
e-
PsaA
PsaD
PsaB
P700
Photosystem I (Thermosynechococcus elongatus)
ATP synthase (Escherichia coli)
3H+
Photosystem II
D1 D2 cp43 cp47 cyt b559
PsbA PsbD PsbC PsbB PsbE PsbF
MSP OEC
PsbL PsbJ PsbK PsbM PsbH PsbI PsbO PsbP
PsbQ PsbR PsbS PsbT PsbU PsbV PsbW PsbX
PsbY PsbZ Psb27 Psb28 Psb28-2
Photosystem I
PsaA PsaB PsaC PsaD PsaE PsaF PsaG PsaH
PsaI PsaJ PsaK PsaL PsaM PsaN PsaO PsaX
Cytochrome b6/f complex
PetB PetD PetA PetC PetL PetM PetN PetG
Photosynthethic electron transport
PC Fd FNR cyt c6
PetE PetF PetH PetJ
F-type ATPase
beta alpha gamma delta epsilon c a b
00195 1/15/14
(c) Kanehisa Laboratories

B

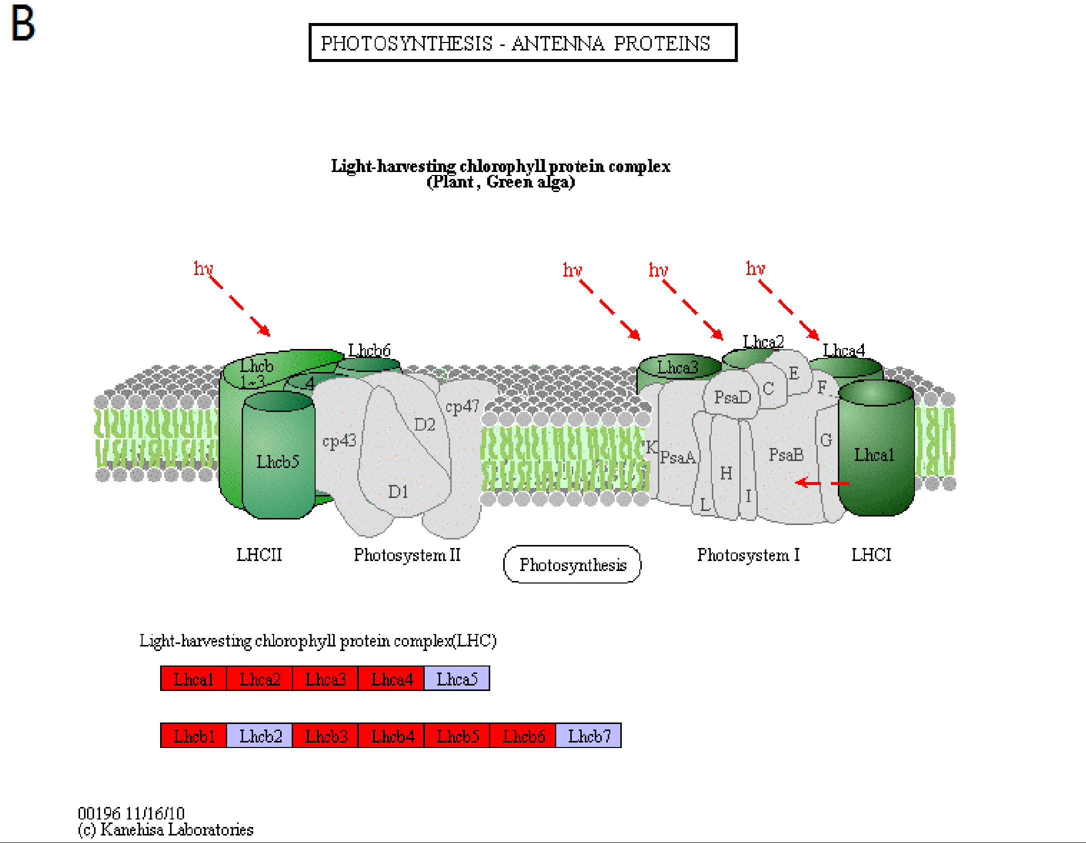
PHOTOSYNTHESIS - ANTENNA PROTEINS
Light-harvesting chlorophyll protein complex
(Plant , Green alga)
hv
Lhcb6
Lhcb 1~3
Lhcb5
cp43
cp47
D2
D1
Lhca3
Lhca2
Lhca4
PsaD
PsaA
PsaB
Lhca1
LHCII
Photosystem II
Photosynthesis
Photosystem I
LHCI
Light-harvesting chlorophyll protein complex(LHC)
Lhca1 Lhca2 Lhca3 Lhca4 Lhca5
Lhcb1 Lhcb2 Lhcb3 Lhcb4 Lhcb5 Lhcb6 Lhcb7
00196 11/16/10
(c) Kanehisa Laboratories

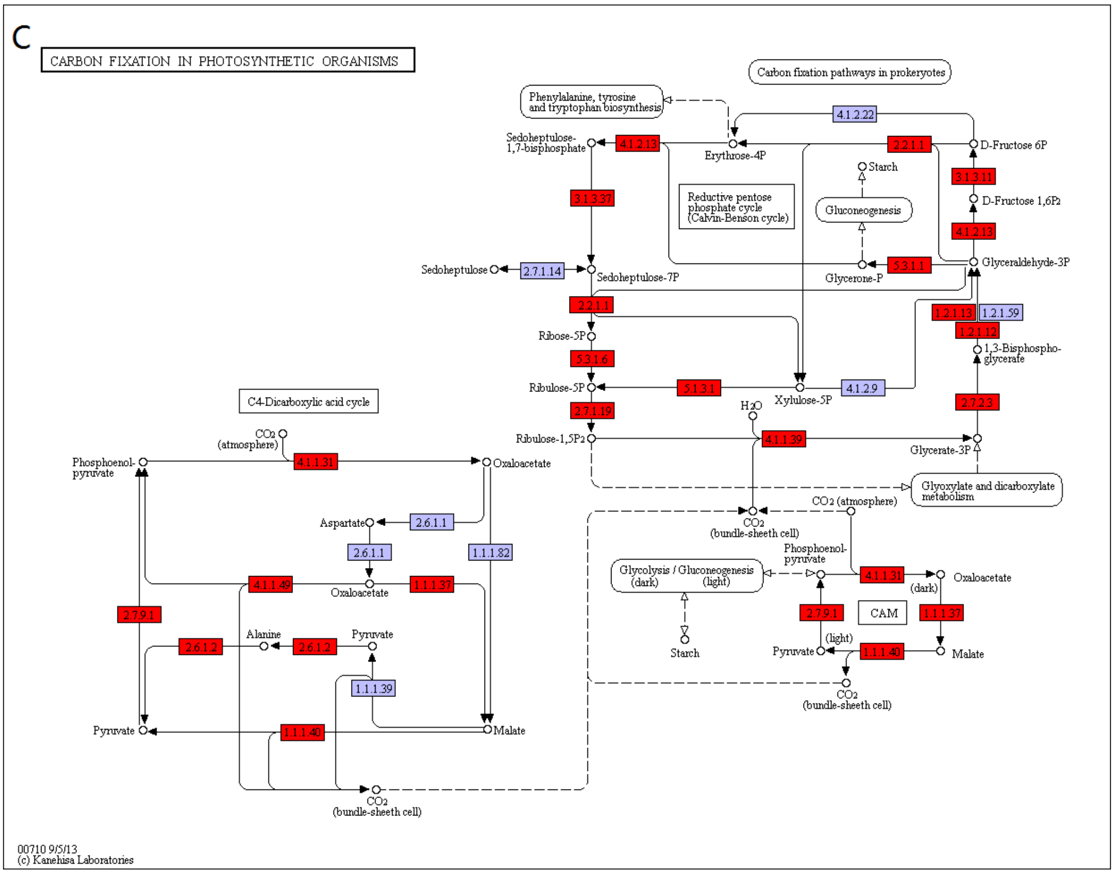

Supplementary figure S8: Unigenes predicted to be involved in the photosynthesis pathway derived from KEGG database (Kanehisa et al., 2012). (A) Photosynthesis. (B) Photosynthesis - antenna proteins. (C) Carbon fixation in photosynthetic organisms. Red box indicates the gene was significantly enriched.

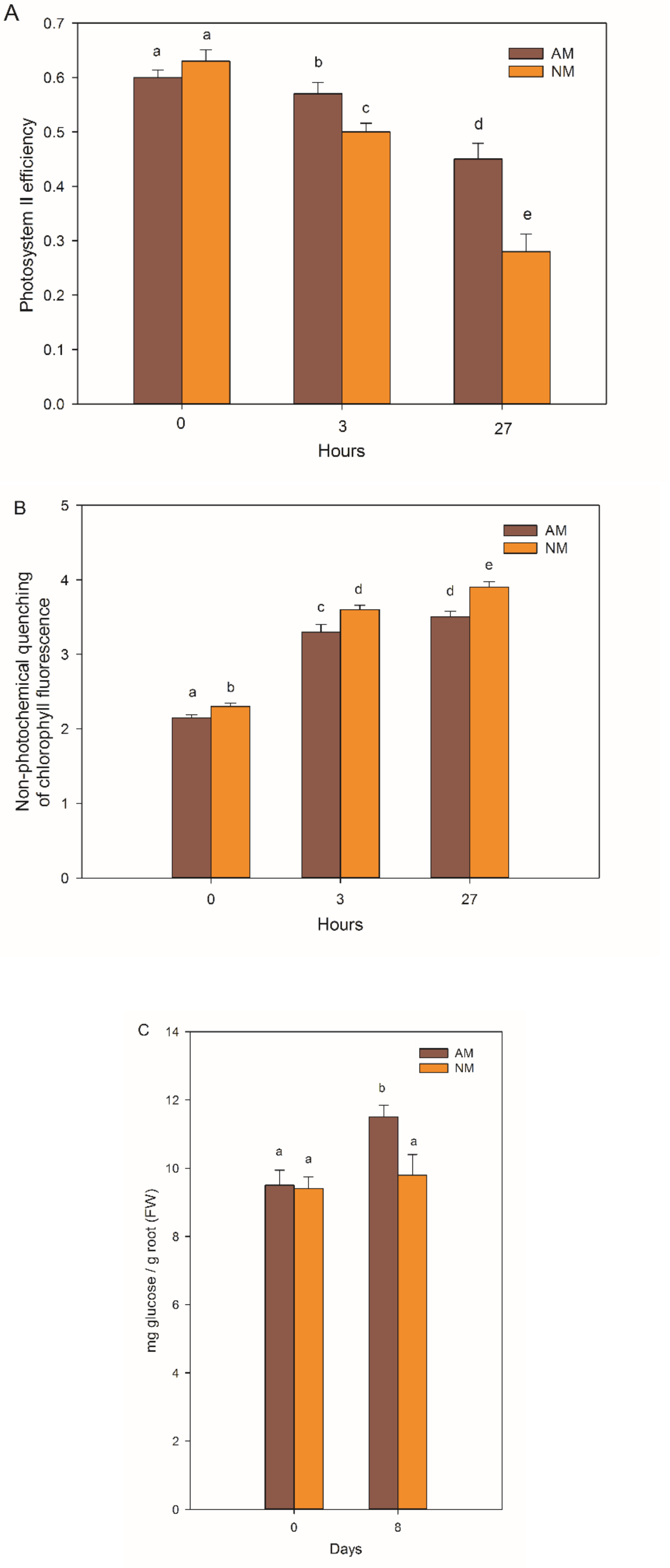
A
Photosystem II efficiency
0.0
0.1
0.2
0.3
0.4
0.5
0.6
0.7
AM
NM
a
a
b
c
d
e
0
3
27
Hours
B
Non-photochemical quenching
of chlorophyll fluorescence
0
1
2
3
4
5
AM
NM
a
b
c
d
d
e
0
3
27
Hours
C
mg glucose / g root (FW)
0
2
4
6
8
10
12
14
AM
NM
a
a
b
a
0
8
Days

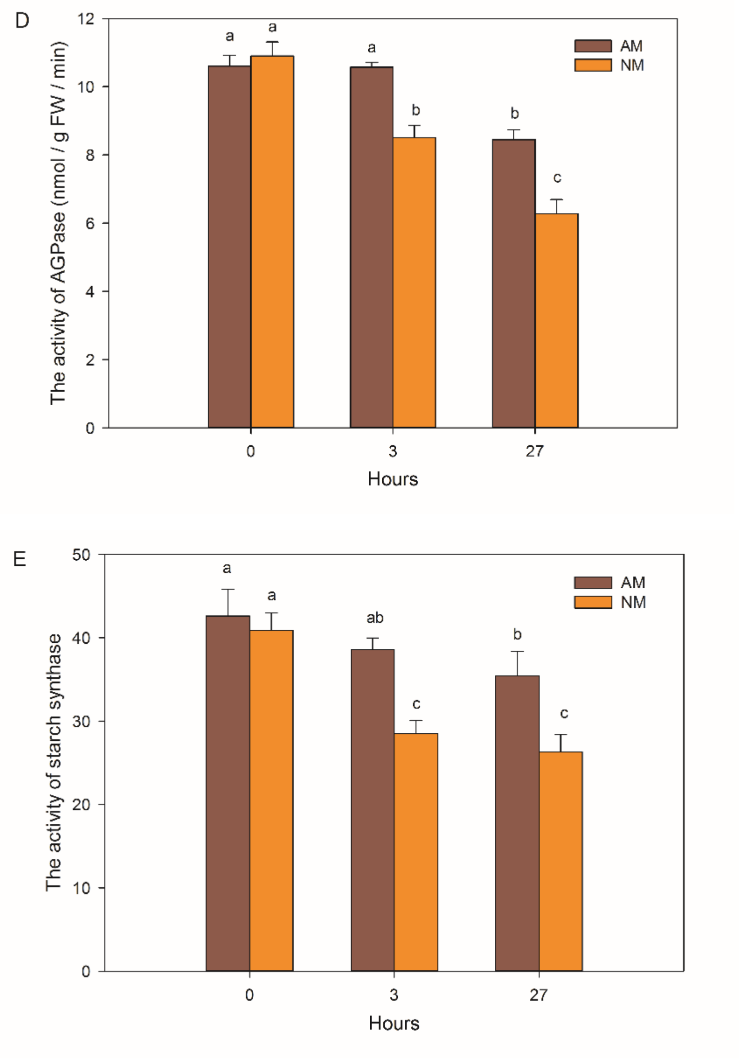

Supplementary figure S9: Effect of stress on the photosynthetic parameters, total root carbohydrate content and activities of starch synthesis-related enzymes of AM and NM Sesbania cannabina plants. (A) Photosystem II efficiency (ΦPSII) values after 3 and 27 hours. (B) Non-photochemical quenching of chlorophyll fluorescence (NPQ) after 3 and 27 hours. (C) Total root carbohydrate content after 8 days. (D) The activity of AGPase after 3 and 27 hours. (E) The activity of starch synthase after 3 and 27 hours. The error bar represents the standard error. Data were analyzed using Duncan's multiple range test. Different letters above the error bars indicate statistical significance at $p < 0.05$.

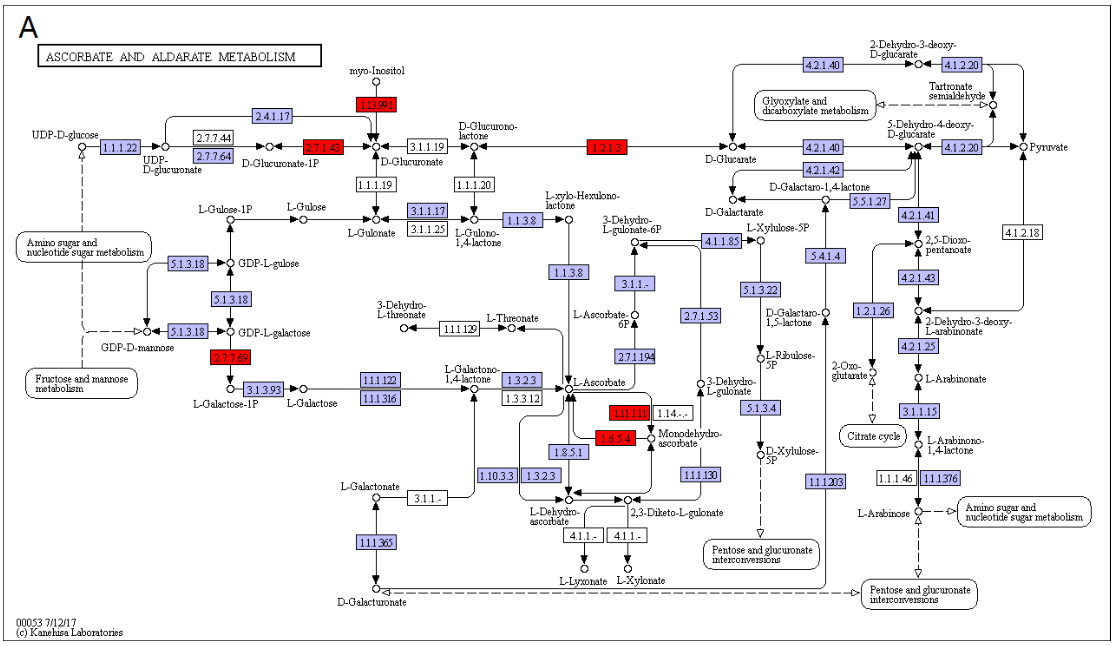

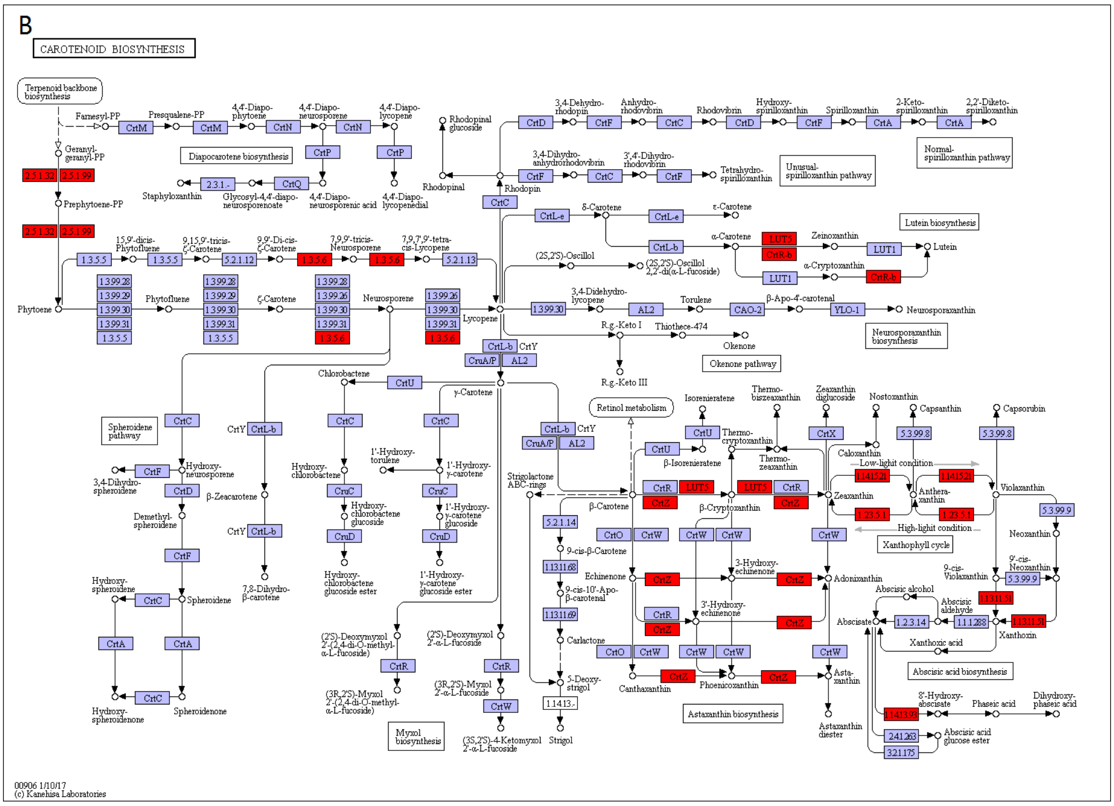

Supplementary figure S10: Unigenes predicted to be involved in the photosynthesis pathway derived from KEGG database (Kanehisa et al. 2012). (A) Ascorbate and aldarate metabolism. (B) Carotenoid biosynthesis. Red box indicates the gene was significantly enriched.

Supplementary tables:

Supplementary table S1: Length frequency and length distribution of transcript and unigene after assembly (See Excel file)**.**

Supplementary table S2: Differential expression of transcripts (See Excel file)**.**

Supplementary table S3: GO enrichment result of bule module (See Excel file)**.**

Supplementary table S4: KEGG pathway enrichment result of bule module (See Excel file)**.**

Supplementary table S5: Network analysis of bule module hub genes.

Supplementary table S6: Network file of Bule module.

Supplementary table S7: Real-time PCR primer information (See Excel file)**.**
